# The experience of living with adolescent idiopathic scoliosis: a qualitative evidence synthesis using meta-ethnography

**DOI:** 10.1186/s12887-023-04183-y

**Published:** 2023-07-22

**Authors:** Erin Hannink, Francine Toye, Meredith Newman, Karen L. Barker

**Affiliations:** 1 Physiotherapy Research Unit, Nuffield Orthopaedic Centre, Oxford University Hospitals NHS Foundation Trust, Windmill Rd., Headington, Oxford, OX3 7HE UK; 2 Nuffield Department of Orthopaedics, Rheumatology and Musculoskeletal Sciences, University of Oxford, Windmill Rd., Headington, Oxford, OX3 7LD UK

**Keywords:** Scoliosis, Qualitative research, Qualitative evidence synthesis, Meta-ethnography, Spinal deformity, Adolescents

## Abstract

**Background:**

Adolescent idiopathic scoliosis (AIS) is a common spinal deformity with physical and psychosocial implications for adolescents. The aim of this qualitative evidence synthesis (QES) was to systematically search for, identify, and synthesise qualitative research in order to improve our understanding of what it is like to live with AIS and to facilitate empathetic and effective healthcare.

**Methods:**

We systematically searched 4 databases (Medline, EMBASE, PsycINFO and CINAHL) and used the 7 phases of meta-ethnography to synthesise qualitative evidence including studies with children and adolescents, and additional viewpoints from parents about the experience of AIS.

**Results:**

We distilled 7 themes. (1) *Diagnosis turned time on its head* revolves around the AIS diagnosis and the uncertainty of the future that accompanied it. (2) *Usual activities no longer the same* explores how activities and participation in everyday life are impacted by AIS. (3) *Hiding my body* describes the pervasive struggle with self-image and appearance. (4) *I want to feel normal again* explores adolescents’ desire to return to ‘normality’ and challenges of feeling different. (5) *Balancing isolation and support* considers the relationships in the adolescents’ lives alongside their feelings of isolation. (6) *Trying to keep control of treatment decisions* explores how adolescents and their parents strive to feel in control. (7) *Fearing surgery yet feeling hopeful* focused on the apprehension and fear around spinal surgery and the beacon of hope it represented.

**Conclusions:**

Our QES contributes to the understanding of the adolescent experience of living with AIS. From our findings, clinicians can better understand the physical and psychosocial obstacles and the challenges faced throughout the journey of AIS to inform their clinical interactions with these patients.

**Supplementary Information:**

The online version contains supplementary material available at 10.1186/s12887-023-04183-y.

## Background

Scoliosis is a structural spinal deformity defined by lateral spinal curvature of 10° or more (using the Cobb method) and spinal rotation [1]. Adolescent idiopathic scoliosis (AIS), which is diagnosed from 10 years old, is the most common type of scoliosis, and there is no known cause. AIS affects 0.5–5.2% of adolescents, predominantly females, aged 10–16 years old [1–3]. Clinical management varies in AIS depending on curve severity and risk factors for curve progression [4]. Typically, the clinical treatment for curvature of 10°-20° is no treatment or “watchful waiting”; spinal bracing and/or physiotherapy-based exercise treatments can be prescribed for those with mild to moderate curvature; and spinal surgery for those with severe (> 45°) curvature [1, 3]. AIS is associated with altered balance, walking and breathing patterns, poorer cardiopulmonary function, lower physical activity, back pain, and low bone mineral density [1, 5–7]. Impacts on function and quality of life increase as curve severity increases. There is no clear relationship in the literature between AIS and overall function, but those with AIS have been shown to be at greater risk of experiencing poorer body image, lower self-esteem and mental ill-health [1, 8, 9] with back pain, lower exercise capacity and treatments such as bracing or surgery affecting quality of life [10]. Overall, long term health-related quality of life can be generally good for people with AIS who undergo surgery or who have conservative treatments, although often worse than those without [1, 11, 12].

Whilst there is qualitative research focused on specific topics such as decision-making and the experience of treatment, the literature exploring what it is like to live with AIS is limited [13]. While individual studies help to describe particular experiences, to date, they have not been integrated into an interpretative qualitative evidence synthesis (QES). QES is a review method, described by Noblit and Hare as having one of two purposes, to summarise and aggregate findings, or to interpret findings in order to generate theories, concepts, models, or “storylines” [13]. We chose the latter purpose to better understand the experience of living with idiopathic scoliosis during the pivotal and impressionable life stage of adolescence. The aim of this QES was to systematically search for, identify, and synthesise qualitative research in order to improve our understanding of what it is like to live with AIS and to facilitate empathetic and effective healthcare.

## Methods

We employed the seven phases of meta-ethnography for synthesising qualitative research findings developed by Noblit and Hare [13] and refined by Toye and colleagues [14, 15]. We used the bespoke eMERGe Reporting Guidance for meta-ethnography [16], and our review was registered on PROSPERO (CRD42021292352).

### Getting started – phase 1

Our original aim for the QES was to explore experiences of physical activity in those living with AIS (prospectively registered with PROSPERO), yet we found no study specifically about this issue in our scoping search. Consequently, we expanded our aim to include any experience of living with AIS as we reasoned this was likely to incorporate experiences of physical activity. We also searched for any existing QES about the experience of living with AIS and found only descriptive reviews. We undertook a meta-ethnography which aims to conceptualise and make ‘a whole into something more than the parts alone imply’ [13].

### Deciding what is relevant – phase 2

We included qualitative studies, published in English, that explored the experience of living with AIS. As this is a young clinical population, we also included parents’ perspectives for additional insight. We excluded studies whose participants had scoliosis secondary to other conditions such as cerebral palsy, juvenile or early-onset (before the age of 10 years) and adult-onset idiopathic scoliosis, as these conditions are associated with a different prognoses, potential interventions and experiences, as well as studies where no in-depth qualitative findings were reported. We followed the mnemonic STARLITE (sampling strategy, type of study, approaches, range of years, inclusion and exclusions, terms used, electronic sources) for reporting literature searches (Table 1) [17]. We searched four bibliographic databases: EMBASE, Medline, PsycINFO and CINAHL. FT and EH screened the titles and abstracts and EH and KB screened the full text of potential studies. Although there is no consensus on quality assessment for qualitative studies, we used the Critical Appraisal Skills Programme (CASP) questions for qualitative research to guide quality appraisal and to help decide whether to include a study in the evidence synthesis [18]. We did not enumerate the CASP questions to determine a hierarchy of quality but used the content of the checklist as a tool for inclusion. If EH and KB did not agree on inclusion, FT was consulted for a final decision.

**Table 1:** STARLITE reporting table

Starlite category	Description
Sampling Strategy	Comprehensive
Type of studies	Qualitative research, fully reported
Approaches	Electronic database with citation snowballing
Range of years	No limit
Limits	None
Inclusion and exclusions	All qualitative studies that explore the experience of adolescent onset idiopathic scoliosis or their parent/carer Exclusions: studies where authentic voice cannot be deciphered from other stakeholder (e.g. health professional or teacher): studies that do not report qualitative findings
Terms used	1 Qualitative Research/ 2 Anthropology/ 3 Focus Groups/ 4 Grounded Theory/ 5 Interview/ 6 1 or 2 or 3 or 4 or 5 7 ethnog*.ti,ab 8 phenomenolog*.ti,ab 9 (qualitative adj5 (theor* or stud* or research or analys*)).ti,ab 10 (hermeneutic* or heidegger* or husserl* or colaizzi* or giorgi* or glaser or strauss or (van and kaam*) or (van and manen) or ricoeur or spiegelberg* or merleau).ti,ab 11 (constant adj3 compar*).ti,ab 12 (grounded adj3 (theor* or stud* or research or analys*)).ti,ab 13 (narrative adj3 analys*).ti,ab 14 (discourse adj3 analys*).ti,ab 15 (conversation adj3 analys*).ti,ab 16 ((lived or life) adj3 experience*).ti,ab 17 ((theoretical or purpose) adj3 sampl*).ti,ab 18 ((field adj note*) or (field adj record*) or fieldnote*).ti,ab 19 (participant* adj3 observ*).ti,ab 20 (action adj research).ti,ab 21 ((digital adj record) or audiorecord*).ti,ab 22 (((co and operative and inquir*) or co-operative) and inquir*).ti,ab 23 ((semi-structured or semistructured or unstructured or structured) adj3 interview*).ti,ab 24 feminis*.ti,ab 25 (humanistic or existential or experiential).ti,ab 26 (social and construct*).ti,ab 27 (poststructural* or post structural* or post-structural*).ti,ab 28 (postmodern* or post modern* or post-modern*).ti,ab 29 'appreciative inquiry'.ti,ab 30 'interpretative phenomenological analysis'.ti,ab 31 (face adj3 interview*).ti,ab 32 ((depth or in-depth) adj3 interview*).ti,ab 33 (abductive adj analys*).ti,ab 34 7 or 8 or 9 or 10 or 11 or 12 or 13 or 14 or 15 or 16 or 17 or 18 or 19 or 20 or 21 or 22 or 23 or 24 or 25 or 26 or 27 or 28 or 29 or 30 or 31 or 32 or 33 35 exp SCOLIOSIS/ 36 scoliosis.ti,ab 37 35 or 36 38 6 and 37 39 34 and 37
Electronic sources	Medline, Embase, PsycINFO, CINAHL

### Reading included studies and determining how they are related – phases 3 & 4

EH read the studies to extract study characteristics contextual information (country of origin, condition and setting, age range, data collection and analytic approach) which would allow readers to compare studies and determine transferability of QES findings. Two reviewers (EH and FT) then read each primary study to identify the ideas or *concepts* from the findings section. Each reviewer wrote these concepts into accessible first-person English to distil the meaning. For example, the phrase “Parents felt it was incumbent upon them to choose the least invasive method of treatment first, and in their minds, that was the brace” [19] became “We chose a brace because we thought it was the least invasive [parent]”. This is a useful process for clarifying meaning and encouraging discussion about differences in interpretation between reviewers. The reviewers compared and discussed the data, intending to add to, rather than agree on a set of concepts for analysis. We divided the study findings by adolescent experience of AIS and parent experience of AIS and repeated the same process for both sets of data. We used NVivo 1.0 software (2020) to aid the development and translation of concepts.

### Translating studies into one another and synthesising translations – phases 5 & 6

Two authors organised the concepts into initial categories and discussed the differences, similarities, and nuances across data. The aim of this process was not to reach consensus, but to distil meaning and to ensure that valuable nuance was not lost in the process of distilling meaning from concept to category. Next, all four authors compared the categories and organised these, again, using the same process of *constant comparison* [20]. Through constant comparison, reviewers explored similarities and differences across and between studies and ideas develop dialectically. Concepts from the child/adolescent were analysed separately from the parent/carer. To assess our confidence in the findings, we employed the four domains of the GRADE-CERQual framework: methodological limitations, relevance, adequacy of data (‘richness and quantity of data’), and coherence (‘consistency across studies’) [21], using the Interactive Summary of Qualitative Findings (iSoQ) online tool [22].

### Expressing the synthesis – phase 7

We used the line of argument approach to create a ‘bigger picture’ of the experience of living with AIS [23]. Using the themes, we developed a conceptual model through discussion between all reviewers and explored our conceptual model in reference to current literature.

## Results

**Fig. 1:**
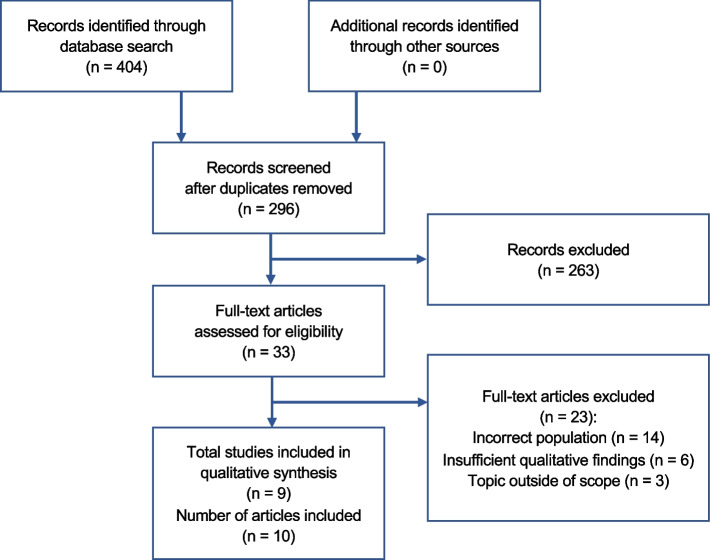
Flow diagram

We screened 296 titles and abstracts and 33 full texts (Fig. 1). We excluded 23 full texts and included data from 10 articles (9 unique studies) [19, 24–32]. The studies incorporated the views from 98 females and 16 males with AIS, aged 10–22 years old, and 16 parents of 14 young women with AIS (Table 2). One study interviewed adolescents and parents [24] and two studies from one trial interviewed adolescents, parents and physiotherapists who delivered an exercise intervention [31, 32]. Studies originated primarily from Europe (UK = 3, Ireland = 1, Sweden = 2, Greece = 1), with North America (USA = 1, Canada = 1) and Asia (Hong Kong = 1) also represented. While the studies all addressed the experience of living with AIS, the focus varied from the experience of undertaking physiotherapy-led exercises [31, 32] to wearing a brace [19, 24, 29] to surgery and surgical recovery [25–28, 30].

**Table 2:** Study characteristics

Author & year	Aim	Country	Condition	Setting	Number	Female (%, n)	Age (y), range	Data collection	Analysis
**Donnelly et al. (2004) **[24]	To explore three aspects of treatment for AIS from the perspective of the patient and family	USA	AIS (brace or surgery)	Tertiary referral center	12 with AIS 10 parents	100%	13–18	Focus groups with patients and group interviews with parents	Content analysis
**Honeyman et al. (2016) **[30]	To provide a balanced, patient-focused, up-to-date, UK-based account of the patient experience of scoliosis surgery	UK	AIS (single stage posterior correction and instrumented fusion surgery)	Surgical hospital	6 with AIS	100%	15–17	Semi-structured interviews	Thematic analysis, interpretive phenomenology
**Law et al. (2016)** [29]	To determine the impact of visual aesthetics on user acceptance and compliance towards the brace	Hong Kong	AIS (20°-30° Cobb with brace)	School screening program and certified prosthetist-orthotist	10 with AIS	100%	12–22	Semi-structured interviews	Grounded theory
**MacCulloch et al. (2009) **[28]	To identify health-specific needs for online information and support for patients with adolescent idiopathic scoliosis who have had or anticipate having spinal surgery	Canada	AIS (post-surgical or anticipating surgery)	Children’s hospital orthopaedic clinic	11 with AIS	82% (*n* = 9)	10–18	Focus groups and one-to-one telephone interviews (*n* = 3); semi-structured interview guide	Content analysis
**Motyer et al. (2022)** [27]	To explore the psychosocial experiences of adolescents with idiopathic scoliosis during the presurgical stage of treatment	Ireland	AIS (pre-surgical)	Orthopaedic department of children’s hospital; tertiary referral center	14 with AIS	71% (*n* = 10)	12–17	Semi-structured interviews	Inductive reflexive thematic analysis
**Rullander et al. (2013) **[26]	To describe adolescents’ narrated experiences of going through scoliosis surgery	Sweden	AIS (post-surgical, ~ 2 years)	Hospital	6 with AIS	67% (n = 4)	15–18	Semi-structured interviews	Content analysis
**Rullander et al. (2017) **[25]	To broaden the scope of adolescents’ experiences of undergoing scoliosis surgery	Sweden	AIS (post-surgical)	Spine centres	37 with AIS	86% (*n* = 32)	12–18	Diary for first 2 weeks of recovery (n = 18), semi-structured interview 6 months post-surgery	Content analysis
**Sapountzi-Krepia et al. (2006) **[19]	To investigate which feelings are created by the bracing experience in children/adolescents with scoliosis and what their opinions of the support provided to them by health-care professionals and by their families	Greece	AIS (brace treatment > 6 months, > 12 h/day)	Outpatient scoliosis clinic in hospital (× 2)	12 with AIS	71% (*n* = 9)	10–16	Semi-structured interviews	Content analysis
**Toye et al. (2016) **[31]** & Williams et al. (2015) **[32]	To explore the feasibility of the trial, including trial recruitment and acceptability of the intervention, participants’ perception of the trial intervention, any issues influencing exercise adherence, and the appropriateness of the chosen trial outcomes	UK	AIS (mild to moderate, participating in physiotherapy exercise programme)	Physiotherapy department of orthopaedic hospital (× 4)	6 with AIS 6 parents; 4 physio-therapists (data not included)	100%	10–16	Semi-structured interviews	Interpretive phenomenological analysis, constant comparative methods

### Quality assessment and confidence in findings

Using the CASP checklist for qualitative research, we agreed that all 10 studies demonstrated adequate quality (Table 3). Five studies did not report the relationship between the researcher and participants, yet deficiency in this category alone was not sufficient to exclude the study. Based on the four domains (methodological limitation, coherence, adequacy of data, and relevance) of the GRADE-CERQual framework, we had moderate to high confidence in all seven themes (Table 4). We did not identify concerns with the methodological limitations as we performed the CASP quality assessment to include the studies which would have flagged methodological flaws that would downgrade our confidence. The coherence of the themes generated minor to very minor concerns when examining the primary data fit with the review finding; the theme around normality was the only theme that we did not downgrade. As this was a review of 10 articles, we found minor concerns with the adequacy of the themes, except for body image and appearance which had particularly rich data. We ascertained minor concerns with the relevance in all themes since the representation of mild disease severity, countries and cultures was limited (Supplementary file 1).

**Table 3:** Study appraisal using the CASP checklist

Authors & year	CASP 1	CASP 2	CASP 3	CASP 4	CASP 5	CASP 6	CASP 7	CASP 8	CASP 9	CASP 10	IN/OUT
Donnelly et al. (2004) [24]	**Y**	**Y**	**Y**	**Y**	**Y**	**Y**	**Y**	**Y**	**Y**	**Y**	**IN**
Honeyman & Davison (2016) [30]	**Y**	**Y**	**Y**	**Y**	**Y**	**Y**	**Y**	**Y**	**Y**	**Y**	**IN**
Law et al. (2016) [29]	**Y**	**Y**	**Y**	**Y**	**Y**	**Y**	**N**	**Y**	**Y**	**Y**	**IN**
Macculloch et al. (2009) [28]	**Y**	**Y**	**Y**	**Y**	**Y**	can't tell	**Y**	**Y**	**Y**	**Y**	**IN**
Motyer et al. (2022) [27]	**Y**	**Y**	**Y**	**Y**	**Y**	can't tell	**Y**	**Y**	**Y**	**Y**	**IN**
Rullander et al. (2013) [26]	**Y**	**Y**	**Y**	can't tell	**Y**	**Y**	**Y**	**Y**	**Y**	**Y**	**IN**
Rullander et al. (2017) [25]	**Y**	**Y**	**Y**	**Y**	can't tell	**Y**	**Y**	**Y**	**Y**	**Y**	**IN**
Sapountzi-Krepia et al. (2006) [19]	**Y**	**Y**	**Y**	**Y**	**Y**	can't tell	**Y**	**Y**	**Y**	**Y**	**IN**
Toye et al. (2016) [31]	**Y**	**Y**	**Y**	**Y**	**Y**	can't tell	**Y**	**Y**	**Y**	**Y**	**IN**
Williams et al. (2015) [32]	**Y**	**Y**	**Y**	**Y**	**Y**	can't tell	**Y**	**Y**	**Y**	**Y**	**IN**

**Table 4:** Theme confidence and representation

	**GRADE-CERQual assessment of confidence**^**a**^	**Theme derived from adolescent/ parent/ physio**	**Donnelly et al. (2004) **[24]	**Honeyman et al. (2016) **[30]	**Law et al. (2016)** [29]	**MacCulloch et al. (2009) **[28]	**Motyer et al. (2022)** [27]	**Rullander et al. (2013) **[26]	**Rullander et al. (2017) **[25]	**Sapountzi-Krepia et al. (2006) **[19]	**Toye et al. (2016) **[31]	**Williams et al. (2015) **[32]
Diagnosis turned time on its head	Moderate	Adolescent & parent		**x**		**x**	**x**				**x**	**x**
Usual activities no longer the same	Moderate	Adolescent & parent	**x**			**x**	**x**	**x**	**x**			**x**
Hiding my body	High	Adolescent & parent	**x**		**x**	**x**	**x**	**x**	**x**	**x**	**x**	
I want to feel normal again	High	Adolescent & parent	**x**		**x**	**x**	**x**		**x**		**x**	**x**
Balancing isolation and support	Moderate	Adolescent	**x**	**x**		**x**	**x**	**x**		**x**	**x**	**x**
Trying to keep control of treatment decisions	Moderate	Adolescent & parent	**x**	**x**	**x**	**x**		**x**		**x**	**x**	**x**
Fearing surgery yet feeling hopeful	Moderate	Adolescent	**x**	**x**		**x**	**x**	**x**	**x**		**x**	**x**

^a^This is a qualitative assessment of confidence made by two reviewers

### Synthesis findings

**Fig. 2:**
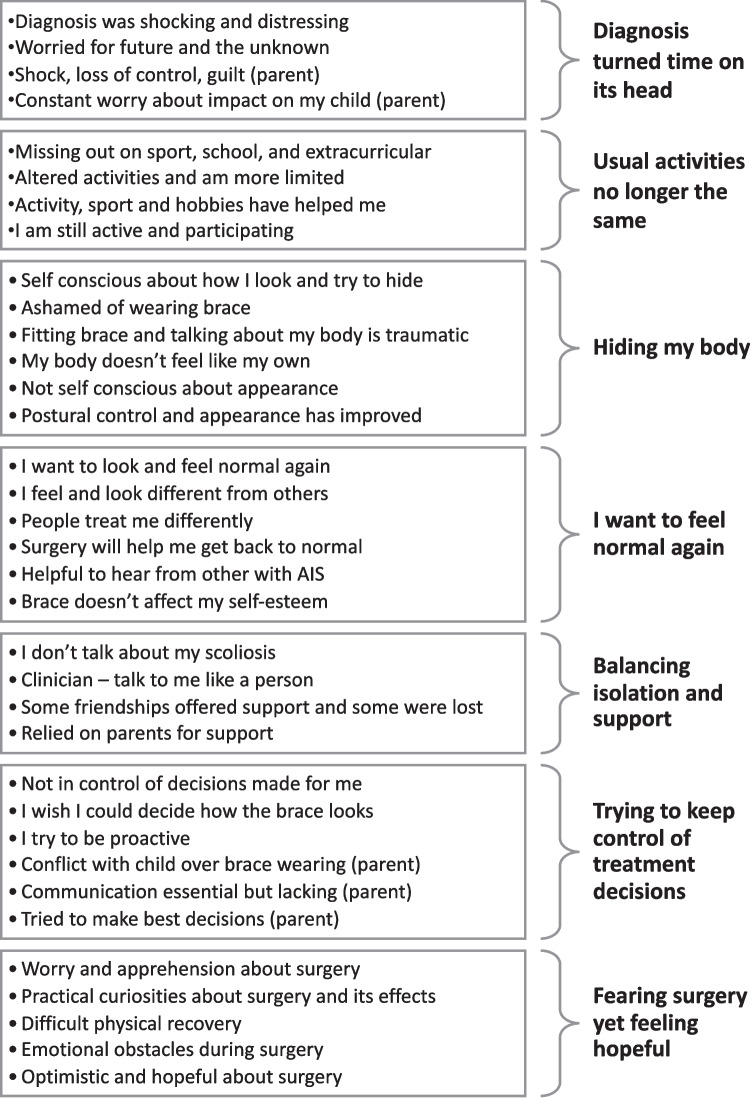
Organisation of 35 categories into 7 themes

From 159 concepts in the primary studies, we formed 35 categories and further distilled to seven final themes: Diagnosis turned time on its head; Usual activities no longer the same; Hiding my body; I want to feel normal again; Balancing isolation and support; Trying to keep control of treatment decisions; and Fearing surgery yet feeling hopeful. Figure 2 shows the organisation of categories into seven themes. Table 4 lists the themes and the studies that support each theme.

#### Diagnosis turned time on its head

This theme revolves around the AIS diagnosis and the uncertainty of the future that accompanied it. The diagnosis was a shock and distressing for some adolescents and their parents [27, 30, 32].

*It’s been quite a rollercoaster ride. I mean one minute I was just healthy, living a good life, and then we found this out, like someone turned the sand timer* (female adolescent) [30].

The point of diagnosis was the first time that some saw or realised they had an abnormal curvature in their spine [28, 31]. This experience was exacerbated when the diagnosis came at a point when curvature was already severe [27, 30].

*That can’t be my spine, is that my spine? It must be somebody else’s spine. They just said straight away that I needed surgery, and… I didn’t take it well* (female adolescent) [30].

Adolescents and parents feared the unknown after diagnosis. Many had questions about the future, in terms of jobs, pregnancy, and ongoing back problems [28, 31, 32]. As one adolescent described, not knowing whether they might need surgery or managed conservative treatment in the future could be very difficult:

*My GP told me I would need surgery or a back brace, so I had prepared myself… and then I got told I didn’t need it as it wasn’t bad enough… he didn’t tell me what options I had to help it, he just said I didn’t need surgery I don’t need a back brace… that was it* (female adolescent) [31].

Some parents noticed their children appeared less worried about the diagnosis and wondered if those living with the condition handled it better than their parents did [31]. Parents felt a sense of loss, guilt, anxiety from not being able to do more, and they were worried about the psychological impact of the condition [31, 32].

*How did I not notice sooner? How did I let it get this far? Is it something I did? I could kick myself for not doing something sooner. I am her parent and can do nothing to make it right* (parent) [32].

#### Usual activities no longer the same

This theme highlights how activities and participation in everyday life were impacted. Usual activities such as going to school, taking part in hobbies, being active with sport, and participating in activities with friends were all affected by scoliosis [24–28, 32]. Adolescents were worried about how surgery might have an impact on all these activities, including getting behind in schoolwork and maintaining friendships during surgical recovery [24–26]. Adolescents self-limited their activities, continued with caution, or even abandoned activities because of scoliosis, the brace and/or surgery [24, 26, 27].

*I had to stop training, and when I could take it up again it felt difficult. The team had grown so much better and I was left behind* (adolescent) [26].

Even when activities were used as a strategy to help through their journey of scoliosis, the activities that composed their daily life were impacted by their scoliosis and/or treatments [24–27]. However, hobbies could also be used as a distraction from impending surgery, and physical activity and sport used as a positive coping mechanism [25, 27, 28].

*Just the fact that I play sport kinda helps me like and just gets my mind off [my scoliosis], I would just totally forget about it there* (female adolescent) [27].

#### Hiding my body

This theme describes the struggle with self-image and hiding the body. Some adolescents were concerned about people seeing their scoliotic curves, and this affected how they went about their life [19, 26, 27].

*I was worried about the brace being visible under my clothes and I didn’t want to go outside the house, not even to have fun* (female adolescent) [19].

Adolescents used strategies to hide their scoliosis, such as readjusting their posture and wearing bigger clothes [27, 31]. Some wanted to hide their brace and found it a source of shame and negativity [19, 27, 29].

*I was ashamed to go out… I was ashamed about wearing the brace at school… I was ashamed when my classmates were looking at me strangely* (female adolescent) [19].

Adolescents and parents both felt that undressing and being asked about their bodies was traumatic, and particularly getting fitted for a brace [24, 31]. As scoliosis became more severe and noticeable, some adolescents looked forward to surgery as a solution [27].

*I could no longer wear the clothing that I liked and it was necessary that I had surgery for me to be happy with my body again* (female adolescent) [27].

After surgery, some adolescents still felt self-conscious about their scar and made efforts to hide it [25, 26, 28]. Conversely, some adolescents were not as affected by their appearance [27].

*[Appearance changes] don’t really bother me too much, like I wouldn’t be 100% happy with how I look, but I think that’s even what most girls are like really* (female adolescent) [27].

#### I want to feel normal again

This theme explores the adolescents desire to return to *normal*. The concept of ‘normality’ was pervasive throughout various aspects of peoples’ lives [24, 25, 27–29, 31, 32]. Feeling different exacerbated loneliness [31, 32].

*I don’t know many people with the same condition, sometimes I feel a bit lonely* (female adolescent) [31, 32].

Adolescents wanted to look and feel normal again, which meant getting back to hobbies and sports and looking ‘straighter’ [25, 27]. In the quest for ‘normality’, there was a sense of resentment and despair about being different and there were constant daily reminders, such as wearing the brace [24, 27–29].

*I ask myself why I was born like this. When I put on the brace again, the physical discomfort further intensifies that feeling* (female adolescent) [29].

Adolescents were concerned that other people would also treat them differently if they knew that they had scoliosis, if they were seen in their brace, or when they were recovering from surgery [24, 28].

*They treat you like, are you sure you can do this: Do you need help carrying that. And it is just kind of annoying* (female adolescent) [24].

Adolescents and parents were comforted by hearing about others who had, or who were going through, a similar experience because it ‘normalised’ their own experience [31, 32]. When peers had scoliosis or wore a brace, it led to feeling less different [29]. One adolescent felt that her self-esteem was not affected because she had a peer who also had a brace, and thus she was not different:

*I didn’t feel that I was inferior to other people when I wore the brace to school. There are some girls who are similar to me, I feel like we are the same* (female adolescent) [29].

#### Balancing isolation and support

This theme examines the relationships in the adolescents’ lives. In some ways adolescents felt they were alone and isolated in their experience of AIS, but they also recognised the crucial role of supportive relationships. Adolescents did not always want to talk about their scoliosis, especially with peers, and this led to feelings of isolation [19, 27, 30].

*Regardless of the support provided, when I was alone I was thinking about it, it concerned me…* (adolescent) [19].

Feelings of isolation were exacerbated by losing touch with friends if they had to drop out of sporting activities, or after a long absence from school recovering from surgery [26, 28].

*I lost contact with [my friends] during the hospital visit, and I still haven’t got them back. I miss them!* (adolescent) [26].

Friendships were a source great support, specifically helping during recovery from surgery [19, 27, 28].

*If I didn’t have the friends who came to visit me, I would have been screwed. I wouldn’t have recovered as fast as I did. I know it sounds cheesy, but support helps you recover faster* (female adolescent) [28].

The relationship with parents was particularly significant. Adolescents expressed an overwhelming appreciation for how much they depended on their parents and received their support during their journey, especially during surgery and the recovery [19, 24, 30].

*I couldn’t let her leave me. I need her, like, so much, it was ridiculous… I just knew I needed me mam, I needed her* (female adolescent) [30].

Relationships between adolescents and their healthcare providers were mixed [24, 31, 32]. Some were able to talk to their doctor and other healthcare providers about their condition, but they did not always feel that there was enough time to build a relationship and to discuss problems and feelings [19, 31].

#### Trying to keep control of treatment decisions

This theme describes the struggle of adolescents and parents to feel in control of treatment decision making following diagnosis. Adolescents with AIS grappled to varying degrees with a sense of lost control over treatment decision-making [19, 24, 26–30]. For those who wore a brace, there was conflict and negotiations with their parents on the number of hours they would realistically wear it [24, 29]. One adolescent wished she had had input, at least in the appearance of the brace:

*I had no choice, I had to wear that hard brace, but it would have made me happier if I was allowed to decide on what could be included (graphics) on the brace, something that I liked. It would have made me feel respected* (female adolescent) [29].

Some were more accepting of ‘big’ decisions, like surgery, being made for them.

*…they just said you have to have surgery. We did not really have a choice but it was fine with me* (female adolescent) [24].

Some gained control by learning about their condition or making a joint decision with their parents [24, 28, 32]. Parents also felt like they lacked control and struggled to make the best decisions by balancing the doctor’s recommendations whilst choosing the least invasive treatment where possible [24, 32].

*…the brace was really, you know, a necessity and so we went ahead and proceeded from that point… we followed doctor’s recommendation on what to do… we more or less trusted the doctor to do the right thing…* (parent) [24].

Parents sought knowledge from others to help them to make decisions [24, 32]. Parents felt that they did not always get the clear communication with healthcare professionals that they needed, and this had an impact on the decision-making process [19, 24, 31]. Some felt that professionals had been ‘cursory’ or dismissive.

*[The doctor] was very cursory… as a child you want someone interested in you rather than only being worried about the angles, saying ‘don’t worry about it everything is fine, off you go’* (parent) [31].

#### Fearing surgery yet feeling hopeful

This theme focused on the experience around spinal surgery and the beacon of hope it represented for some. Surgery, apprehension of surgery, and recovery from surgery were all associated with fear [25–28, 30–32]. Adolescents described anxiety and fear leading up to surgery, concentrating on unlikely detrimental effects and complications [26, 27, 30].

*I was scared of being paralyzed and not being able to walk again, to sort of have to be bound to a wheelchair* (adolescent) [26].

Adolescents described a long and difficult physical recovery from surgery, from the pain and immobility to nausea and constipation from medications [24–28, 30]. They were surprised by how difficult and energy-consuming every task was during the first few days of recovery [25–27].

*It seems such a little thing to have to do but it was so much effort like to get on a toilet and then walk across the room... it was a lot of pain and work and energy* (female adolescent) [30].

Along with the physical recovery from surgery, there were also emotional obstacles including stress, irritability, frustration, feelings of regret and overall feeling ‘so darned helpless’ [25, 26, 28, 30]. Beyond the negative aspects of surgery, for some, it represented a beacon of hope and symbolised the end of a journey [27]. Adolescents felt optimistic that surgery would do more than just straighten their back but ‘would just be really good and helpful, and make me feel good, better about myself as well’ [27]. Finally, surgery represented the opportunity to be *normal* again [27, 28].

*Once I get the surgery done I feel like I’ll be able to get back to normal* (female adolescent) [27].

### Conceptual model

**Fig. 3:**
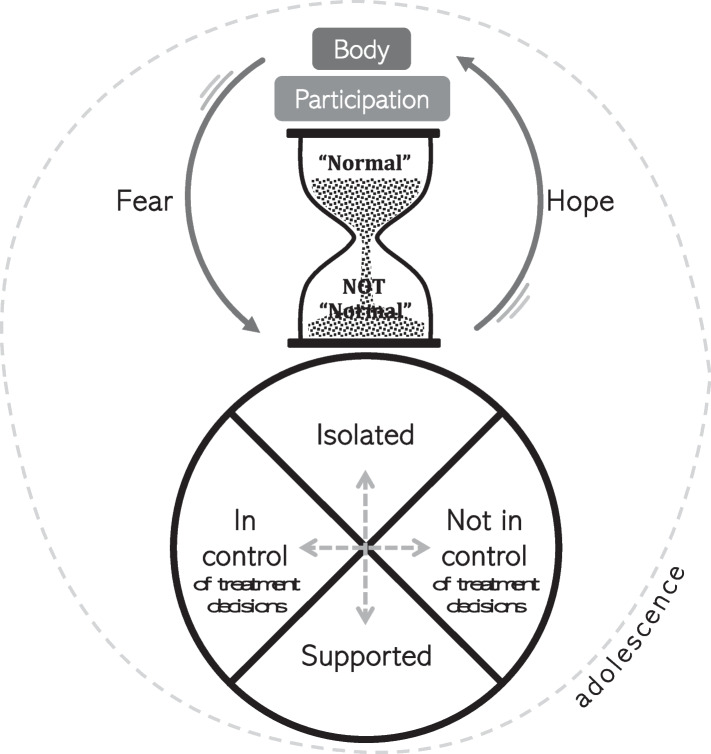
Conceptual model

Figure 3 incorporates the essence of the themes into a conceptual model. The turning of the hourglass exemplifies the sudden disruption of time (theme: Diagnosis turned time on its head) and disruption of normal life with the diagnosis of scoliosis. The sand falling into the bottom chamber represents *normal* becoming *not normal* (theme: I want to feel normal again). Above the hourglass are ordinary parts of life that were once *normal* that have now been disrupted: the body and participation. The body has become something that is hidden (theme: Hiding my body), and participation in everyday activities have been impacted (theme: Usual activities no longer the same). Fear and hope (theme: Fearing surgery yet feeling hopeful) flank the hourglass and are onset by the disruption of time. Feelings of fear come with aspects of *normal* slipping away, such as impending surgery, and feelings of hope come with the prospect that *not normal* may become *normal* again, such as feeling optimistic about successful treatment. Prominently at the bottom of the model is a circle that depicts the tensions between being in control and not in control of treatment decisions (theme: Trying to keep control of treatment decisions) and being isolated and being supported (theme: Feeling alone, feeling supported). The proximity and location of each side of these tensions also illustrate the interaction between all four poles. All of this is taking place with adolescence as a backdrop, a transitional and influential stage of life.

## Discussion

This evidence synthesis is the first to use a meta-ethnographic approach to gain a deeper understanding of the experience of living with AIS. The QES highlights adolescents’ need for ‘normality’ and the impact of an uncertain future. It also elucidates the feelings of shock and disruption at diagnosis, and the apprehension and hope that comes for those who require surgery. A desire to hide their body and the limitations on school and sport activities were also prevalent themes during this formative stage of life. The earlier scoping review by Essex et al. 2022 [33] similarly reported findings of shock and distress at diagnosis and experiences around body image and perception. However, this earlier review focused on summarising views on treatments such as bracing and surgery to inform future research; whereas our QES sought to take the analysis a stage further by developing new concepts to better understand the experience of living with AIS.

The synthesis findings and conceptual model point to the diagnosis of scoliosis as a turning point that disrupts the usual flow of time and kicks off a cascade of experiences expressed in the QES themes. The accounts and feelings conveyed by adolescents and parents about the disruption and subsequent impacts after the diagnosis fit Bury’s theoretical concept of a ‘biographical disruption’, which is a rupture in the fabric of everyday life that results in the disruption of narratives about the future and the person’s identity [34]. This appears acutely influential in the period of adolescence in which AIS takes place. The idea of wanting to be *normal* is part of adolescent identity development and is a pervasive theme during adolescence whether the person has AIS or not. Adolescents with AIS have a more visible struggle with amplified physical changes and disruptions to their life: from asymmetries in their body, to a bulky spinal brace, to their medical absences in school, to their post-surgical scar. In some ways the struggle resonates with the journey of other conditions such as cancer in adolescence [35]. Being *different* from peers and the strain on relationships with peers goes hand in hand with the desire to be *normal* [36]. The findings from our meta-ethnography portray adolescents striving to be *normal* compared to typically developing peers and striving to get back to their own sense of *normal*. In our conceptual model, we illustrate how *normal* gets turned on its head with the disruption of an adolescent’s scoliosis diagnosis.

Related to a desire to obtain ‘normality’, the themes around self-image and activity examine the impact of AIS on ordinary parts of an adolescent’s life. It seems that the struggle to adjust to body changes during growth is amplified due to visible differences in spinal shape and posture, potential scarring post surgery and/or use of braces that alter body image and appearance in this and could link to lower self-esteem. Bracing interventions with loss of control over appearance could also be a source of conflict and stress. Concerns about appearance were on a spectrum, where less severe spinal curvature could go unnoticed by the adolescent and by others, and sometimes the first X-ray was the earliest recognition of their abnormally curved spine. A struggle with negative body image was apparent in our findings and is in line with quantitative literature that reports high prevalence of body image disorders in people with AIS [9]. Psychological factors such as lower self-image and lower mental health alongside the magnitude of change in spinal alignment are reported risk factors for experiencing back pain and lower quality of life in adolescents with AIS [37]. The impact of AIS on specific hobbies, physical activity and sports varied, but was felt by adolescents in all stages of their AIS journey. Interference and disturbance to normal activities can affect adolescents’ identity development [38] which contributes to the important theme around activity that emerged in this synthesis. The themes around self-image and activity are interconnected and both also linked to positive social feedback, feelings of control and fitting in among their adolescent peer group [39, 40]. Connections between more positive self-image and higher levels of activity in AIS irrespective of curve severity are also reported in quantitative studies [41].

The synthesis of the ideas from our included studies captures adolescents at different stages along the journey of AIS starting at diagnosis, diverging based on the progression of spinal curvature severity and conservative treatments (physiotherapy and bracing), and for some, culminating with surgery and post-surgical recovery. The experiences we synthesised suggest a period for some pre-diagnosis where AIS has minimal impact on the adolescent’s life, and then a disruption with diagnosis. Along this journey, findings suggested some experience of frustration with the period of “watchful waiting.” While the medicalised term of monitoring was not explicitly used by adolescents and parents, their frustration with the concept emerges when they describe the period of uncertainty and worry following diagnosis and frustrations with perceived relative inaction and loss of control, whilst waiting potentially for disease progression. In this context for some surgery could be positive, a hopeful action to resolve visible differences, address uncertainty and halt progression These experiences contrast with experiences of other childhood conditions, where disease pathophysiology is known, outcomes more predictable and/or where interventions are available and commence on diagnosis. Since each individual study primarily focused on one time point or experience connected to different AIS disease severities and management options, the QES enabled us to shed a broader light on different stages and thus the experience of living with AIS.

The addition of the parent viewpoint was important for this clinical group since adolescents are becoming more independent from 10–18 years old yet still rely on their parents in the decision-making process and for physical and emotional support. Both adolescents and parents expressed a shock at the diagnosis and fear of an unknown future, but parents expanded on their feelings of guilt and powerlessness to help their child [31, 32]. How children handled the diagnosis and lived with the condition exceeded parents’ expectations, which could be attributed to adolescents’ internalisation or their stage of AIS, experiencing minimal impact or seeing hope at the end of the tunnel with surgery [27, 28, 31, 32].

### Methodological comments

Our QES used the seven phases of meta-ethnography which set it apart from Essex et al. (2022) [33] who performed a scoping review and narrative synthesis of qualitative evidence that applied broader inclusion criteria incorporating studies that we excluded [42–44] because the authors did not report in-depth qualitative findings. Our review aims also differed; while Essex et al. (2022) [33] focused on summarising the literature among topics related to AIS to identify shortcomings and direction for future research, our QES sought to develop new concepts to better understand the experience of living with AIS.

### Limitations

Our QES and the literature in general has a limited male perspective on living with the condition. Although more females are diagnosed with AIS, it is important that males are included, as their experience may be qualitatively different. Quantitative data show differences in self-reported outcome measures around mental health, pain and self-image between males and females [45]. Of the 9 studies, 5 studies included boys, making up 14% of the total sample. It is likely that there are differences in the gendered experiences of living with AIS, and future qualitative research in AIS should address this to ensure that gendered nuances that might have an impact on healthcare are considered: future studies should consider a spectrum of gendered experiences. Although international, our findings were drawn from seven countries, none of which were low-income, and future studies might include different international perspectives. It is not uncommon to find that qualitative research does not report on ethnicity or socio-economic diversity of the included sample and this can have an important bearing on lived experience. We would recommend that future qualitative research report these details in their samples so that readers can make a judgement about transferability of findings outside the context, and so that reviewers can ascertain gaps in experiential knowledge.

We did not include PPIE (patient and public involvement and engagement) members or stakeholders to contribute to the synthesis of the papers and develop the conceptual model; one of our research team has experience of undergoing spinal surgery for AIS in her past, but only represents her personal experience. We acknowledge that wider PPIE is important to ensure that the review is relevant and meaningful to the people affected by the condition as well as stakeholders using the review to inform policy or practice [46]. Constraints in time and budget made this challenging and we did not include them in Step 7 as recommended by Noblit and Hare. This would have added experiential knowledge of the condition to the interpretation, but these may differ from the original source quotes of the research participants in the primary papers. We have ameliorated this by working closely with the national scoliosis charity on PPIE work in developing further research.

The lack of primary research also means that our QES cannot capture the experience of living with AIS into adulthood. Although onset is during adolescence, people continue to live with scoliosis, sometimes with instrumentation along their spine, for the rest of their lives, or with the prospect of future surgery. Furthermore, recent research suggests scoliosis increases lifetime risks of adverse cardiovascular events and osteoporosis [47, 48]. Additionally, only one study in our review included people with mild to moderate scoliosis, therefore the perspectives of this group need further investigation e.g., experiences of medical monitoring, uncertainty about prognosis, waiting for progression. Lastly, the confidence in our findings based on the GRADE-CERQual tool are an interpretation, and other reviewers might assess this differently. For example, adequacy is a qualitative judgement about the “richness” and/or “adequacy” of the data supporting a QES finding and “there are no fixed rules regarding what constitutes sufficiently rich data or an adequate quantity of data” [49].

### Reflexive statement

The strength of qualitative research lies in its interpretation and each researcher will code and analyse data through their own unique interpretive lens. Being open to constructive criticism and being able to reflect on the impact of our personal viewpoint (reflexivity), is therefore an important facet of qualitative research rigour. The aim of collaboration is to challenge and develop analytic decisions, rather than to reach consensus. Our team had a range of professional and personal experience of scoliosis and felt free to discuss, suggest, and challenge each other. EH is a physiotherapy researcher with a particular interest in spinal alignment. FT is a qualitative researcher and anthropologist who has undergone spinal fusion for AIS. MN is a physiotherapist with experience treating people with scoliosis and has worked alongside young people with scoliosis and their families as PPIE. KB is a physiotherapist with experience of treating patients with AIS and an academic researching the evaluation physiotherapy and rehabilitation interventions.

### Clinical implications

This evidence synthesis can help clinicians better understand the experience of adolescents with AIS throughout the journey of the condition. The findings also elucidate the variability of lived experience of AIS which is critical for clinicians to recognise. Findings from both adolescents and parents highlight the importance of clear and compassionate communication and shared decision-making for treatment e.g., around bracing. They emphasise the need to pay attention to monitoring as a treatment strategy, to the impact of changes in body shape and activities. This has implications for policy and practice as greater awareness of the impact that diagnosis and treatment has on both adolescents and parents, may prompt providers and clinicians to consider greater use of psychological and therapeutic interventions to support mental health and participation in activities for adolescents and families at this time.

## Conclusions

Our meta-ethnography contributes to the understanding of the adolescent experience of living with AIS. Our conceptual model illustrates the impact of AIS from the moment of disruption to the impact on everyday life to the desire to feel *normal* again. From our findings, we hope clinicians can better understand the physical and psychosocial obstacles and the evolution of these challenges throughout the journey of AIS to inform their clinical interactions with these patients.

## Supplementary Information

**Additional file 1.**

## Data Availability

The datasets used and/or analysed during the current study are available from the corresponding author on reasonable request.

## Abbreviations

AIS: Adolescent idiopathic scoliosis
QES: Qualitative evidence synthesis
STARLITE: Sampling strategy, type of study, approaches, range of years, inclusion and exclusions, terms used, electronic sources
CASP: Critical Appraisal Skills Programme
GRADE-CERQual: Grading of Recommendations Assessment, Development and Evaluation Confidence in Evidence from Reviews of Qualitative research
PPIE: Patient and public involvement and engagement
