# Supplementary material for: The experience of living with adolescent idiopathic scoliosis: a qualitative evidence synthesis using meta-ethnography
Source: BMC Pediatr. 2023 Jul 22;23:373. doi: 10.1186/s12887-023-04183-y (PMC10362777; doi:10.1186/s12887-023-04183-y)
Supplement: Supplementary file 1 — Additional file 1. [file 12887_2023_4183_MOESM1_ESM.pdf]

Experiences of people living with AIS
Summary of Qualitative Findings Table

Review question

What are the experiences of people living with adolescent-onset idiopathic scoliosis?

#	Summarised review finding	GRADE-CERQual Assessment of confidence	Explanation of GRADE-CERQual Assessment	References
1	Diagnosis of scoliosis turned the sand timer. This theme revolves around the AIS diagnosis and the uncertainty of the future that accompanied it.	Moderate confidence	No/Very minor concerns regarding methodological limitations, Minor concerns regarding coherence, Minor concerns regarding adequacy, and Minor concerns regarding relevance	Motyer et al. 2021; Toye et al. 2016; Macculloch et al. 2009; Honeyman & Davison 2016; Williams et al. 2015;
2	Normal activities no longer normal. This theme explores how activities and participation in everyday life were impacted.	Moderate confidence	No/Very minor concerns regarding methodological limitations, Minor concerns regarding coherence, Minor concerns regarding adequacy, and Minor concerns regarding relevance	Motyer et al. 2021; Donnelly et al. 2004; Rullander et al. 2017; Rullander et al. 2013; Macculloch et al. 2009; Williams et al. 2015;
3	Hiding my body. This theme describes the struggle with self-image and hiding the body.	High confidence	No/Very minor concerns regarding methodological limitations, Minor concerns regarding coherence, No/Very minor concerns regarding adequacy, and Minor concerns regarding relevance	Law D et al. 2017; Motyer et al. 2021; Donnelly et al. 2004; Rullander et al. 2017; Toye et al. 2016; Sapountzi-Krepia et al. 2006; Rullander et al. 2013; Macculloch et al. 2009;
4	I want to feel normal again. This theme explores the adolescents desire to return to normal.	High confidence	No/Very minor concerns regarding methodological limitations, No/Very minor concerns regarding coherence, Minor concerns regarding adequacy, and Minor concerns regarding relevance	Law D et al. 2017; Motyer et al. 2021; Donnelly et al. 2004; Rullander et al. 2017; Toye et al. 2016; Macculloch et al. 2009; Williams et al. 2015;
5	Balancing isolation and support. This theme examines the relationships in the adolescents' lives.	Moderate confidence	No/Very minor concerns regarding methodological limitations, Minor concerns regarding coherence, Minor concerns regarding	Motyer et al. 2021; Donnelly et al. 2004; Toye et al. 2016; Sapountzi-Krepia et al. 2006; Rullander et al. 2013; Macculloch et al. 2009; Honeyman & Davison 2016; Williams et al. 2015;

#	Summarised review finding	GRADE-CERQual Assessment of confidence	Explanation of GRADE-CERQual Assessment	References
			adequacy, and Minor concerns regarding relevance	
6	Trying to keep control. This theme describes of the struggle to feel in control of decision-making following diagnosis.	Moderate confidence	No/Very minor concerns regarding methodological limitations, Minor concerns regarding coherence, Minor concerns regarding adequacy, and Minor concerns regarding relevance	Law D et al. 2017; Donnelly et al. 2004; Toye et al. 2016; Sapountzi-Krepia et al. 2006; Rullander et al. 2013; Macculloch et al. 2009; Honeyman & Davison 2016; Williams et al. 2015;
7	Fearing surgery yet feeling hopeful. This theme focused on the experience around spinal surgery and the beacon of hope it represented for some.	Moderate confidence	No/Very minor concerns regarding methodological limitations, Minor concerns regarding coherence, Minor concerns regarding adequacy, and Minor concerns regarding relevance	Motyer et al. 2021; Donnelly et al. 2004; Rullander et al. 2017; Toye et al. 2016; Rullander et al. 2013; Macculloch et al. 2009; Honeyman & Davison 2016; Williams et al. 2015;

Evidence Profile Table

#	Summarised review finding	Methodological limitations	Coherence	Adequacy	Relevance	GRADE-CERQual assessment of confidence	References
1	Diagnosis of scoliosis turned the sand timer. This theme revolves around the AIS diagnosis and the uncertainty of the future that accompanied it.	No/Very minor concerns<br><br>**Explanation:** No studies have more than one CASP category that was not explicitly reported.	Minor concerns<br><br>**Explanation:** Minor concerns regarding coherence because because of the varying primary data fitting with the review finding.	Minor concerns<br><br>**Explanation:** Minor concerns regarding adequacy because there were only 5 studies contributing to this review finding.	Minor concerns<br><br>**Explanation:** Minor concerns regarding relevance because the studies included were from UK, Canada and Ireland where the diagnostic process may differ.	Moderate confidence<br><br>**Explanation:** No/Very minor concerns regarding methodological limitations, Minor concerns regarding coherence, Minor concerns regarding adequacy, and Minor concerns regarding relevance	Honeyman & Davison 2016; Macculloch et al. 2009; Motyer et al. 2021; Toye et al. 2016; Williams et al. 2015;
2	Normal activities no longer normal. This theme explores how activities and participation in everyday life were impacted.	No/Very minor concerns<br><br>**Explanation:** No studies have more than one CASP category that was not explicitly reported.	Minor concerns<br><br>**Explanation:** Minor concerns regarding coherence because because of the varying primary data fitting with the review finding.	Minor concerns<br><br>**Explanation:** Minor concerns regarding adequacy because only 6 studies discussed this review finding.	Minor concerns<br><br>**Explanation:** Minor concerns regarding relevance because only Western countries are included in this theme.	Moderate confidence<br><br>**Explanation:** No/Very minor concerns regarding methodological limitations, Minor concerns regarding coherence, Minor concerns regarding adequacy, and	Donnelly et al. 2004; Macculloch et al. 2009; Motyer et al. 2021; Rullander et al. 2013; Rullander et al. 2017; Williams et al. 2015;

#	Summarised review finding	Methodological limitations	Coherence	Adequacy	Relevance	GRADE-CERQual assessment of confidence	References
						Minor concerns regarding relevance	
3	Hiding my body. This theme describes the struggle with self-image and hiding the body.	No/Very minor concerns<br><br>**Explanation:** No studies have more than one CASP category that was not explicitly reported	Minor concerns<br><br>**Explanation:** Minor concerns regarding coherence because of the outlier data around the theme presented in a couple studies.	No/Very minor concerns<br><br>**Explanation:** All studies with this theme provide rich data around this review finding.	Minor concerns<br><br>**Explanation:** Minor concerns regarding relevance because of the representation of the countries/cultures from these studies is limited.	High confidence<br><br>**Explanation:** No/Very minor concerns regarding methodological limitations, Minor concerns regarding coherence, No/Very minor concerns regarding adequacy, and Minor concerns regarding relevance	Donnelly et al. 2004; Law D et al. 2017; Macculloch et al. 2009; Motyer et al. 2021; Rullander et al. 2013; Rullander et al. 2017; Sapountzi-Krepia et al. 2006; Toye et al. 2016;
4	I want to feel normal again. This theme explores the adolescents desire to return to normal.	No/Very minor concerns<br><br>**Explanation:** No studies have more than one CASP category that was not explicitly reported.	No/Very minor concerns<br><br>**Explanation:** The extracted data fit with this review finding.	Minor concerns<br><br>**Explanation:** Minor concerns regarding adequacy because of the data and limited number of studies supporting this review finding.	Minor concerns<br><br>**Explanation:** Minor concerns regarding relevance because of the representation of the countries/cultures from these studies is limited.	High confidence<br><br>**Explanation:** No/Very minor concerns regarding methodological limitations, No/Very minor concerns regarding coherence,	Donnelly et al. 2004; Law D et al. 2017; Macculloch et al. 2009; Motyer et al. 2021; Rullander et al. 2017; Toye et al. 2016; Williams et al. 2015;

#	Summarised review finding	Methodological limitations	Coherence	Adequacy	Relevance	GRADE-CERQual assessment of confidence	References
						Minor concerns regarding adequacy, and Minor concerns regarding relevance	
5	Balancing isolation and support. This theme examines the relationships in the adolescents' lives.	No/Very minor concerns<br><br>**Explanation:** No studies have more than one CASP category that was not explicitly reported.	Minor concerns<br><br>**Explanation:** Minor concerns regarding coherence because because of the varying primary data fitting with the review finding.	Minor concerns<br><br>**Explanation:** Minor concerns regarding adequacy because of the data and limited number of studies supporting this review finding.	Minor concerns<br><br>**Explanation:** Minor concerns regarding relevance because all studies come from Western countries.	Moderate confidence<br><br>**Explanation:** No/Very minor concerns regarding methodological limitations, Minor concerns regarding coherence, Minor concerns regarding adequacy, and Minor concerns regarding relevance	Donnelly et al. 2004; Honeyman & Davison 2016; Macculloch et al. 2009; Motyer et al. 2021; Rullander et al. 2013; Sapountzi-Krepia et al. 2006; Toye et al. 2016; Williams et al. 2015;
6	Trying to keep control. This theme describes of the struggle to feel in control of decision-making following diagnosis.	No/Very minor concerns<br><br>**Explanation:** No studies have more than one CASP category that was not explicitly reported.	Minor concerns<br><br>**Explanation:** Minor concerns regarding coherence because because of the varying primary data	Minor concerns<br><br>**Explanation:** Minor concerns regarding adequacy because of the data and limited number of studies	Minor concerns<br><br>**Explanation:** Minor concerns regarding relevance because of the representation of the countries/cultures	Moderate confidence<br><br>**Explanation:** No/Very minor concerns regarding methodological limitations, Minor concerns regarding	Donnelly et al. 2004; Honeyman & Davison 2016; Law D et al. 2017; Macculloch et al. 2009; Rullander et al. 2013; Sapountzi-Krepia et al. 2006; Toye et al. 2016;

#	Summarised review finding	Methodological limitations	Coherence	Adequacy	Relevance	GRADE-CERQual assessment of confidence	References
			fitting with the review finding.	supporting this review finding.	from these studies is limited.	coherence, Minor concerns regarding adequacy, and Minor concerns regarding relevance	Williams et al. 2015;
7	Fearing surgery yet feeling hopeful. This theme focused on the experience around spinal surgery and the beacon of hope it represented for some.	No/Very minor concerns<br><br>**Explanation:** No studies have more than one CASP category that was not explicitly reported.	Minor concerns<br><br>**Explanation:** Minor concerns regarding coherence because because of the varying primary data fitting with the review finding.	Minor concerns<br><br>**Explanation:** Minor concerns regarding adequacy because of the data and limited number of studies supporting this review finding.	Minor concerns<br><br>**Explanation:** Minor concerns regarding relevance because of the representation of the countries/cultures from these studies is limited.	Moderate confidence<br><br>**Explanation:** No/Very minor concerns regarding methodological limitations, Minor concerns regarding coherence, Minor concerns regarding adequacy, and Minor concerns regarding relevance	Donnelly et al. 2004; Honeyman & Davison 2016; Macculloch et al. 2009; Motyer et al. 2021; Rullander et al. 2013; Rullander et al. 2017; Toye et al. 2016; Williams et al. 2015;
